# National HIV Testing Day — June 27, 2015

**Published:** 2015-06-26

National HIV Testing Day, June 27, promotes the importance of testing in detecting, treating, and preventing human immunodeficiency virus (HIV) infection. HIV testing is the essential entry point to a continuum of prevention, health care, and social services that improve the quality of life and the length of survival for persons with HIV (1). Recent findings show significantly greater health benefits for persons who start antiretroviral therapy (ART) earlier (2). Persons with HIV who receive appropriate treatment, monitoring, and health care also reduce their chances of transmitting HIV to others (3). The key to HIV treatment, care, and prevention is learning one’s status through testing.

In 2011, an estimated 1.2 million persons were living with HIV infection in the United States; an estimated 86% were diagnosed with HIV, 40% were engaged in HIV medical care, 37% were prescribed ART, and 30% achieved viral suppression (1). This issue of *MMWR* includes a report presenting estimates of the prevalence of diagnosed and undiagnosed HIV infections by state during 2008–2012.

Additional information on National HIV Testing Day is available at http://www.cdc.gov/features/HIVtesting. Basic testing information for consumers is available at http://www.cdc.gov/hiv/basics/testing.html.

Additional information on HIV testing for health professionals is available at http://www.cdc.gov/hiv/testing. CDC’s guidelines for HIV testing of serum and plasma specimens are available at http://www.cdc.gov/hiv/testing/laboratorytests.html.
